# Space dynamic target tracking method based on five-frame difference and Deepsort

**DOI:** 10.1038/s41598-024-56623-z

**Published:** 2024-03-12

**Authors:** Cheng Huang, Quanli Zeng, Fangyu Xiong, Jiazhong Xu

**Affiliations:** Heilongjiang Provincial Key Laboratory of Complex Intelligent System and Integration, School of Automation, Harbin University of Science and Technology, Harbin, 150080 China

**Keywords:** Aerospace engineering, Engineering

## Abstract

For the problem of space dynamic target tracking with occlusion, this paper proposes an online tracking method based on the combination between the five-frame difference and Deepsort (Simple Online and Realtime Tracking with a Deep Association Metric), which is to achieve the identification first and then tracking of the dynamic target. First of all, according to three-frame difference, the five-frame difference is improved, and through the integration with ViBe (Visual Background Extraction), the accuracy and anti-interference ability are enhanced; Secondly, the YOLOv5s (You Look Only Once) is improved using preprocessing of DWT (Discrete Wavelet Transformation) and injecting GAM (Global Attention Module), which is considered as the detector for Deepsort to solve the missing in occlusion, and the real-time and accuracy can be strengthened; Lastly, simulation results show that the proposed space dynamic target tracking can keep stable to track all dynamic targets under the background interference and occlusion, the tracking precision is improved to 93.88%. Furthermore, there is a combination with the physical depth camera D435i, experiments on target dynamics show the effectiveness and superiority of the proposed recognition and tracking algorithm in the face of strong light and occlusion.

## Introduction

As space technology develops gradually, more and more countries will be able to enter space technology research, and thus space resources will also become increasingly tight, it is necessary for current space powers to effectively avoid space accidents as much as possible. At present, all the countries that can launch space targets are striving to build and refine their space target database, which includes all kinds of targets, such as the satellites operating on low, medium, and high orbits, decommissioned satellites, large space debris and various planets in space celestial systems. For the space target monitoring system, one of the significant tasks is to monitor the mentioned-above space targets, through this system, the perception area can be accurately recognized, the operation state can be tracked stably, and the operation data can also be obtained to perform a comprehensive analysis. Once there are abnormal operation data, orbit-changing movement, and security treatment from other objects under the operation environment, the monitoring system will feedback warning information promptly, which is an effective way to avoid space collision.

The key to the success of space-based monitoring tasks lies in the autonomous recognition and tracking technology, and most of the related technologies have the problems of slow speed, low accuracy, slow calculation speed of tracking, and easy loss of targets, which seriously affects the execution of monitoring tasks. Additionally, the space background with illumination variation, deformation, motion blur and occlusion also constraint on the task of monitoring from Ref.^1^. With the rapid development of CNN (Convolutional Neural Network), compared with traditional target recognition and tracking, CNN-based recognition and tracking have the performance advantages of high accuracy and timeliness, which can effectively deal with the above problems.

Identifying changing or moving regions in a camera's field of view is a fundamental pre-processing step in computer vision and video processing. To date, many motion and change detection algorithms have been developed that perform well in certain types of videos, but most are sensitive to sudden lighting changes (e.g. bright light), environmental conditions (e.g. night), background/camera motion, shadow, and camouflage effects.

Since Girshick et al.^2^ proposed the Region based Convolutional Neural Network (R-CNN) algorithm in 2014, the use of CNN in the field of target recognition has received more attention and research. However, there are still problems of redundant feature extraction and image distortion caused by scaling, which have an impact on recognition accuracy and speed. Furthermore, Girshick^3^ proposed an improved version of the algorithm Fast R-CNN, but Fast R-CNN still needs to train the Selective Search (SS) algorithm separately, so it is still non-end-to-end. Since then, Ren et al.^4^ have further improved the algorithm for this problem and proposed Faster R-CNN based on the region candidate network. Nevertheless, CNN relies on the target dataset, and when the number of datasets is insufficient, it may cause overfitting and poor generalization ability. And it still cannot meet the performance requirements of real-time recognition and has the disadvantage of a large amount of calculation.

YOLO summarizes target detection as a regression to achieve end-to-end training and detection. Due to its good trade-off of speed-accuracy, it has been in a leading position in the field of target detection in recent years and has been successfully researched, improved and applied to many different fields.

Therefore, in response to the shortcomings of the two-stage algorithm described above, Redmon et al.^5^ proposed a unified regression-based real-time object recognition algorithm YOLO (You Only Look Once). The YOLOv2^6^ based on Darknet-19, and YOLOv3^7^ by referring to the residual network structure were proposed but each grid can only predict one target category and a poor recognition effect still exists. Since then, Alexey et al.^8^ have used CSPDarknet-53 as the Backbone in pursuit of a higher-performance recognition and used the Mish function to propose YOLOv4. Meanwhile, Ultralytics^9^ proposed the YOLOv5 algorithm with more speed and accuracy. To my best knowledge, Lee et al.^10^ conducted YOLOv5-based learning to improve the object detection accuracy of $32 \times 32$ pixels or less and analyzed the YOLOv5s model-based inference performance. As a result of the analysis, it was confirmed that the inference performance of the customized dataset that performed qualitative augmentation using the classical image processing method was improved. Mahendrakar et al.^11^ combined a machine vision feature recognition that is YOLOv5 and localization algorithm and an artificial potential field guidance law to enable small chaser spacecraft to safely approach and capture a rotating, non-cooperative resident space object in on-orbit servicing or active debris removal applications. Reference^12^ conducted a comparative study of various version YOLO to a conclusion as which algorithm would be the best and effective for the detection of objects.

However, the occlusion caused by the position of the light source and the black background of the universe exerts a great influence on vision-based recognition. For the occlusion, Ref.^13^ studied a tracking algorithm in a spatiotemporal context (STC) framework, where the occlusion is detected from Average Peak to Correlation Energy (APCE)-based mechanism of response map between consecutive frames. And Ref.^14^ proposed a moving object detection method based on improved inter-frame difference, which mainly combines the characteristics of the image to determine the appropriate number of frames, and the difference operation is performed. After the operation, every pixel will be processed by classification, and the noise interference can be filtered by a method of thresholds finally. But the ‘bilateral thick outline’ and ‘holes’ are the challenge for the inter-frame difference. And thus, Ref.^15^. studied a moving target detection system combining a three-frame difference and Gaussian model, which adopts inter-frame difference and threshold segmentation to extract the moving region in the image, and its experiment results show that noise immunity robustness can be achieved. Still, it's hard to get out of the predicament of the ‘holes’ and ‘ghosts’ when changing velocity for moving targets in three-frame difference. For the ‘ghosts’, Ref.^16^ proposed an improved ViBe-based motion target detection method, which mainly applies the improved ViBe-based algorithm and the adaptive dynamic thresholding five-frame difference algorithm into the detection of the motion target and the binary foreground images obtained by these two methods with logical ‘or’ operation to eliminate the shadow disturbance of the motion target. And Ref.^17^ also indicated that the five frame difference has a certain anti-interference ability in the face of complex backgrounds, such as the separation of foreground and background. Compared with the three-frame difference, due to more considered frames, the ‘holes’ from some targets with the similar color can be avoided more likely and the sudden changes in light can also be dealt with. Comparatively, unlike the seven-frame difference, more frames will bring about more time of calculation and poor real-time. Nevertheless, the ViBe is seen as a non-parametric estimation method, whose threshold setting is a key point, different targets will be different under different scenarios. In addition, for the dynamic target recognition, there is another way of research thought that is obtaining the target dynamic information as much as possible by background information to build the model of the image. Reference^18^ proposed a novel moving object detection algorithm with dynamic mode decomposition and YOLOv5, the moving object buried in the dynamic foreground and reconstructed images or videos were recognized by Yolov5. Agrawal et al.^19^ a novel approach to detect moving objects from static scenes using a single stationary camera, which mainly utilizes the statistical background model Gaussian Mixture Model (GMM) to generate the binary mask, and at this stage, the model parameters were adjusted aiming to update background model pixel-wise. Additionally, Wang et al.^20^ through another logical thought meaning that random selection of static frames and adding into every other frame to be free from algorithmic dependence on the background and decrease the influence caused by changes in the background. Meanwhile, Ding et al.^21^ proposed foreground–background merging to alleviate background bias, which mainly deliberately composes the moving foreground region of the selected video onto the static background of others. By leveraging the semantic consistency between the original clips and the fused ones, the model focuses more on the motion patterns. Still, transient stationary states of dynamic objects or transient motion of static objects can bring about potential false or missed detection for background image-based methods. Besides, the above background methods of Gaussian-based parameters estimation face the high cost of parameter calculation and it is hard to satisfy the space real-time as well. The practical space environment is not exclusively a single object appearing in the region of vision-based recognition, but a variety of different objects. At the same time, it is extremely possible for the occlusions among them, which is a big challenge for recognition and tracking algorithms. Tlig et al.^22^ proposed a Multi-Object tracking method, which primarily combines the radar and image to measure data based on the Kalman filtering. Among them, the GMM detecting foreground, and the filter step is performed to refine the detection results. Undeniably, the GMM does possess the advantage of posterior knowledge, but it is still difficult to alleviate the nuisance of too many parameters. Reference^23^ proposed a real-time tracking system, which takes the YOLOv5s into object detection and uses the SORT algorithm for executive tracking aiming at keeping all air and ground objects within the field of view of the monocular camera fixed to the UAV. And yet there will be potential target loss when the matching is not achieved between the predicted position of the tracked target and the IOU of the detection frame. Furthermore, Deepsort can alleviate this problem by using a more reliable metric instead of the correlation metric in SORT. Obviously, under the multi-objects, the Deepsort has more research and application value. Against possible micro-drones with malicious intent to attack, Ref.^24^ proposed an online multiple object tracking strategy based on YOLOv5 and the Deepsort tracker to early warning detect and track rogue mini-UAVs in restricted areas. For the low recognition rate of mini-UAV, Ying et al.^25^ proposed a combination of Deepsort detection and tracking algorithm to avoid frame loss with video detection. Reference^26^ developed two new models about granulated RCNN and multi-class Deepsort and took the video as input. The former can extract regions of interest by incorporating the unique concept of granulation in a deep convolutional neural network, and the latter searching for the association of objects with trajectories was restricted only within the same categories. This increased the performance in multi-class tracking. References^27–30^ apply some good new novelties in the Deepsort in all kinds of field including agriculture, transportation, marine object detection and tracking and son on. And some comparisons are shown in these literatures to indicate that the Deepsort is widely used and effective. From the mentioned statement of Deepsort, multi-object tracking is a clear research trend. However, to my best knowledge, this algorithm involves Mahalanobis distance, which will be invalid when the target motion has a large uncertainty, while the cosine distance could be valid more. With the cascades between the two metrics, both of the selections of weight coefficient and threshold super-parameter of metric will change according to a specific dataset.

In response, an online tracking method for identifying dynamic targets first and then tracking them is proposed, and this paper makes the following contributions to the above description to achieve further effective improvements in spatial multi-target recognition and tracking in the best possible way:

1. A five-frame difference incorporating ‘or’ and ‘and’ logical operations is proposed to reach effective suppression of background interference, ‘holes’ and ‘ghosts’. And given that five-frame difference has the potential problem of slow background update, the fusion is made. The background model is updated in time to extract as much of the complete moving target area as possible, by fusing the five-frame difference with ViBe-based background subtraction to compensate for potential ‘holes’ in the five-frame difference.
2. DWT and GAM are added to optimize the Deepsort detector, with the aim of solving the target loss in case of target occlusion and meeting the demand for real-time accuracy in dynamic target online tracking.
3. A large number of real environment simulation experiments are conducted on the improved algorithm, such as background of dimness, bright light, and target continuous occlusion.

## Problem statement

**Figure 1:**
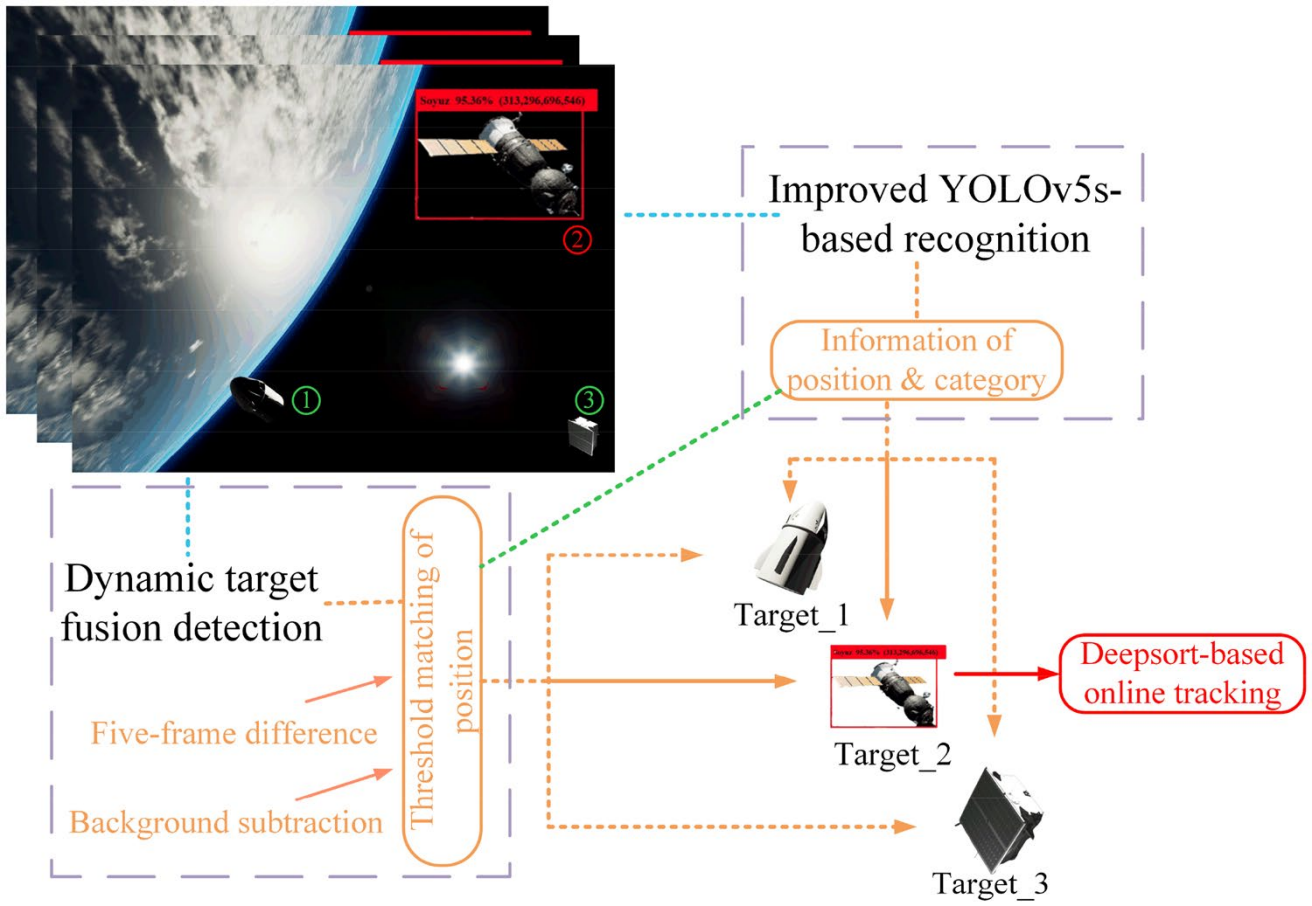
Diagram of autonomous recognition and tracking of dynamic spatial targets.

In the task of space dynamic target tracking, the proposed methods are divided into two steps of dynamic target identification and tracking whose structure is shown in Fig. 1. Specifically, the three spatial images in the figure are depicted as on-board online real-time multi-images. The dynamic target identification phase uses the five-frame difference and the ViBe-based background subtraction to design a space dynamic target detection method that identifies dynamic targets from the static background of sequential image frames and utilizes the results as the task target during the tracking process; Taking the improved YOLOv5s recognition module as the Deepsort detector to design an efficient tracking method for the identified dynamic targets. In Fig. 1, under the parallel operation of the dynamic fusion detection module and the improved YOLOv5s-based recognition module, the two modules' data is interoperable and multiple types of information within the image will be obtained, such as centroid position, category, and region. In addition, based on the proposed fusion method, dynamic targets will be presented from the static background of the sequential image frames. Combining these two parallel modules, the position information will be matched by the threshold to achieve the effect of identifying the dynamic targets. As can be clearly seen in the figure with the red display box, the visualization interface will add easy-to-identify display boxes to the dynamic target boundaries after the identification, and provide online real-time tracking of the target's real-time trajectory.

As shown in Fig. 1, the occlusion caused by the position of the light source and the black background of the universe can constrain the image-based recognition algorithm, a problem that is well illustrated by target object number 1 in the figure. This is reflected by the fact that one side of the object can be detected facing the light source, while its shadow side is the same black as the cosmic background, a phenomenon that can clearly be seen as a recognition where the target is occluded or mutilated. Further, the real-time online process is time-varying, with differences in occlusion at different moments, if the shaded part becomes recognizable after a certain period, and it is easy to fall into the misconception of a new target. Therefore, some problems that are difficult to adequately simulate during ground experiments will arise throughout the recognition, matching, and tracking, which is a greater challenge for the existing algorithms.

**Figure 2:**
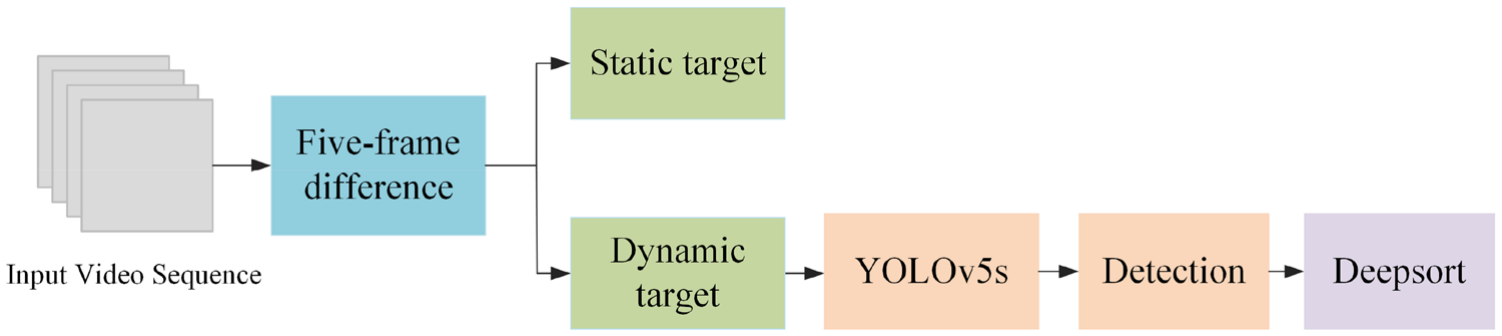
The process of each module.

As shown in Fig. 2, the main process of this paper is to first separate the dynamic targets from the multi-target spatial environment through the designed five-frame difference, and then use the improved YOLOv5s to detect from the image frames under the dark background, and then match the detection results based on YOLOv5s the prediction results of Deepsort, and finally achieve online tracking of dynamic targets.

### Frame-difference

**Figure 3:**
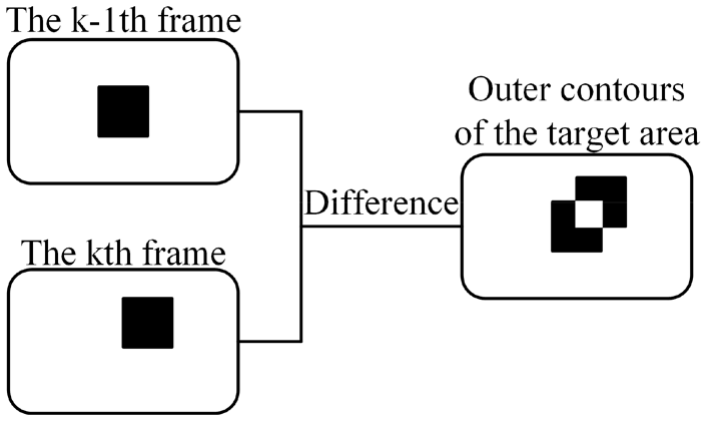
Diagram of inter-frame difference.

Image-based recognition techniques are subject to background interference. Generally, the cosmic background is dark with high-brightness spatial sun rays, and the inter-frame difference method can be targeted to improve the algorithm in this context. The main advantages of this method are its low complexity and simplicity, its low dependence on background information, and its resistance to interference from light intensity. The details of the inter-frame difference are shown in Fig. 3. In short, the method is a simple analysis and judgment of dynamic targets using the absolute value of the grey scale difference, and the formula corresponding to Fig. 3 is shown in (1).

1 $$D_{k} (x,y) = \left| {I_{k} (x,y) - I_{{k{ - }1}} (x,y)} \right|$$

The (1) can be expressed in two main sides, on the one hand as inter-frames, represented by $I_{k}$ and $I_{k - 1}$. On the other hand, the two-dimensional pixel points within a single frame are represented by $(x,y)$. Finally, $D_{k} (x,y)$ is then binarized to obtain the dynamic target region.

**Figure 4:**
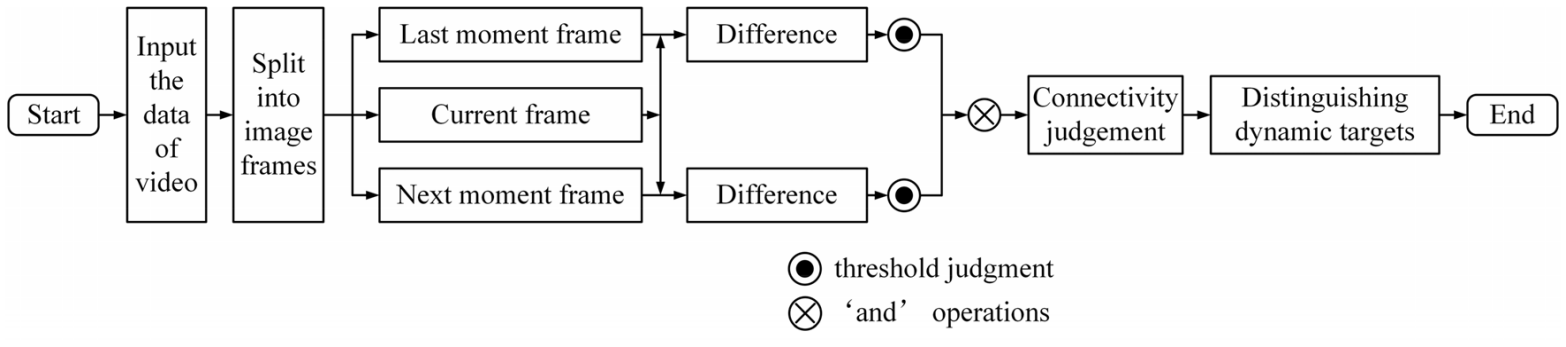
The process of three-frame difference.

Nevertheless, the ‘bilateral thick outline’ and the ‘holes’ inside the target are obvious shortcomings of this method, which will make it difficult to extract the content of the target^31^. One more frame is added to this method, which means a three-frame difference, the mentioned-above problem can be optimized to a certain extent by the idea of inter-frame difference, as shown in Fig. 4.

The main thought is to calculate the two values of the difference between the three frames and perform a logical ‘and’ operation on them. The false detection of a dynamic target is effectively mitigated like a shadowed surface such as that shown in Fig. 1 for target number 1. However, while the background interference can be effectively suppressed, the target is a dynamic time-varying object and the speed of motion will affect the performance, such as a ‘hole’ if the motion velocity shifts to be slow, otherwise a ‘ghost’.

### ViBe-based background subtraction

From another point of view, the recognition of dynamic targets can be indirectly obtained by the background information. Building a good background image model based on background subtraction can be considered a core tool for this idea. In fact, background subtraction is achieved by constructing a static background model and using the grey scale difference between the sequence image and the background model image to achieve the analysis of the threshold, and the accuracy is mainly dependent on the background image. Further, if the static target is in motion or the dynamic target is static in the background image, there will be a lot of missed and wrong detection. In addition, in background modeling, the Gaussian modeling-based parametric estimation requires a model design before parameter estimation, whereas the non-parametric estimation-based background subtraction method does not require assumptions about the overall distribution, and only uses a priori knowledge to make statistical and analytical judgments directly, thus avoiding a large amount of parameter calculation and achieving an effective speed-up in comparison.

ViBe-based background subtraction represents a non-parametric estimation whose main advantage is the adaptive updating of the background. The method is mainly based on Euclidean distance for model building. Since other pixels in the neighborhood of a pixel in an image influence that pixel, the ViBe-based background subtraction creates a sample set for all pixel points on the image, each sample set is used to store the historical pixel values and domain pixel values of a pixel point. For background modeling, each pixel point consists of $N$ randomly selected pixel points within the domain of that pixel point.

**Figure 5:**
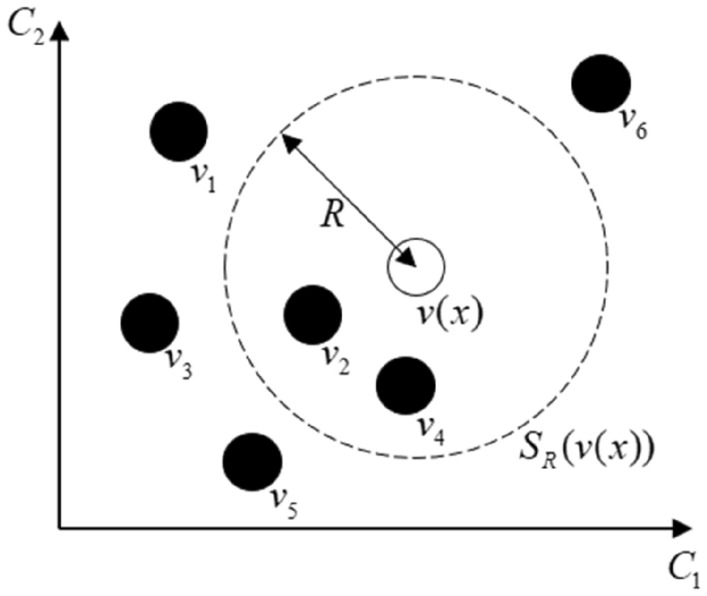
Pixel distribution in Euclidean space.

Set the value of the $x$th pixel in Euclidean space on the image be $v(x)$, randomly select background sampling point with size of $N$ for modeling within the field $N_{G} (x)$ of the pixel $x$, and the value of the background sampling point indexed $i$ in Euclidean space is $v_{i}$, $M(x) = \{ v_{1} ,v_{2} ,...,v_{N} \}$ represents the sample set composed of all background sampling points, and $S_{R} (V(x))$ represents the 2-dimensional Euclidean space with the pixel $x$ as the center and $R$ as the radius. According to Fig. 5, if the pixel $x$ satisfies the condition of (2), the pixel $x$ can be regarded as the background pixel.

2 $$\# \{ S_{R} (V(x)) \cap \{ v_{1} ,v_{2} ,...,v_{N} \} \} \ge \#_{\min }$$

where $\# \{ S_{R} (V(x)) \cap \{ v_{1} ,v_{2} ,...,v_{N} \} \}$ is seen as the intersection between the $M(x)$ and $S_{R} (V(x))$, $\#_{\min }$ is the threshold, which is generally $\#_{\min } = 2$ and $N = 20$, $R = 20$. The threshold $\#_{\min }$ and parameter *R* are a standard invariance value, which are set with many times of experiment. Details about them can be found in^32^.

Since the update mechanism of the basic background subtraction is such that dynamic target pixel points inappropriate for filling the background image, this can cause a deadlock where if the background point is mistakenly detected as a dynamic target point in the initial frame, the pixel point is always remembered as a dynamic target point. If an update mechanism is used where both background and dynamic target points can be used to fill the background image, the missed detection may occur when the object is moving slowly, so the ViBe-based background subtraction combines the two update mechanisms and uses a memoryless update mechanism.

Assume that a background point has a probability ${1 \mathord{\left/ {\vphantom {1 N}} \right. \kern-0pt} N}$ to update the background model of itself or a pixel point in the domain when the number of a dynamic target point is remembered as a background point reaches a threshold $T$, the dynamic target point can be updated as a background point, at this time a sample point is randomly selected within $N_{G} (x)$ for background update, the practical random update mechanism can maintain the probability of any point in the sample set being used to update the background model at a time $t$ is ${1 \mathord{\left/ {\vphantom {1 N}} \right. \kern-0pt} N}$, the retained probability is ${{(N - 1)} \mathord{\left/ {\vphantom {{(N - 1)} N}} \right. \kern-0pt} N}$. From this, the probability of being retained at other times can be derived $P(t,t + dt)$ as follows:

3 $$P(t,t + dt) = \left( {\frac{N - 1}{N}} \right)^{(t + dt) - t}$$

4 $$P(t,t + dt) = e^{{ - \ln (\frac{N - 1}{N})dt}}$$

From the above equations, the retained probability of samples is irrelevant to the time $t$. And so, the retention time of the sample values in the background model is therefore guaranteed to decay smoothly according to an exponential scale.

Nevertheless, the detection results of the ViBe-based background subtraction will depend on the setting of the threshold. Specifically, when the threshold is set high, some useful changes are easily ignored and valid information is easily lost; conversely, noise such as slight environmental changes in the background will be detected and interfere with the detection results, so the setting of the threshold in the non-parametric estimation is a key point.

### Online real-time tracking algorithm based on deep association metrics

As can be seen from the schematic diagram in Fig. 1, three spacecraft are present in the sequence diagram on the left. Given the actual space environment, there are multiple types and numbers of space targets. In turn, a multi-target-based tracking algorithm to design a dynamic tracking method is an important factor to be considered in most of these scenarios, where the main idea is to seek the target area from the sequence image through the target detector, and then use feature extraction and threshold judgment to achieve sequence number assignment for each target. In addition, agents in the space environment are in high-speed motion, and online and real-time requirements are also more demanding compared to the ground, and the more prominent idea is to use Kalman filtering for prediction, and then use the Hungarian algorithm to achieve IOU matching^33^. However, this method is prone to the loss of effective feature information, which in turn leads to the loss of the target, and there is also a logical drawback that the target needs to be retracted after it is lost, and its corresponding serial number is prone to frequent changes.

To improve the robustness of false detection, the Deepsort^34^ uses a new update mechanism in the Kalman filtering: a newly detected target point in a frame is marked as tentative, and if the match is successful for $N$ consecutive frames, the line of target points in these $N{ + }1$ frames is considered as a new trajectory.

The target is assumed to move in an eight-dimensional space $(x,y,\gamma ,h,\dot{x},\dot{y},\dot{\gamma },\dot{h})$, where $(x,y,\gamma ,h)$ respectively represents the horizontal coordinates of the target centroid, the vertical coordinates, the aspect ratio of the target area, and the height of the target, and $(\dot{x},\dot{y},\dot{\gamma },\dot{h})$ represents the rate of change of the first four parameters in the image sequence, respectively. The a priori estimate of the mean $x^{\prime}_{t}$ at the time $t$ can be obtained from the state transfer matrix $F$ and the mean posterior estimate $x_{t - 1}$ at the time $t - 1$:

5 $$x^{\prime}_{t} = \left[ {\begin{array}{*{20}c} x \\ y \\ \gamma \\ h \\ {\dot{x}} \\ {\dot{y}} \\ {\dot{\gamma }} \\ {\dot{h}} \\ \end{array} } \right]_{t} = Fx_{t - 1} = \left[ {\begin{array}{*{20}c} 1 & 0 & 0 & 0 & {dt} & 0 & 0 & 0 \\ 0 & 1 & 0 & 0 & 0 & {dt} & 0 & 0 \\ 0 & 0 & 1 & 0 & 0 & 0 & {dt} & 0 \\ 0 & 0 & 0 & 1 & 0 & 0 & 0 & {dt} \\ 0 & 0 & 0 & 0 & 1 & 0 & 0 & 0 \\ 0 & 0 & 0 & 0 & 0 & 1 & 0 & 0 \\ 0 & 0 & 0 & 0 & 0 & 0 & 1 & 0 \\ 0 & 0 & 0 & 0 & 0 & 0 & 0 & 1 \\ \end{array} } \right]\left[ {\begin{array}{*{20}c} x \\ y \\ \gamma \\ h \\ {\dot{x}} \\ {\dot{y}} \\ {\dot{\gamma }} \\ {\dot{h}} \\ \end{array} } \right]_{t - 1}$$

In (5), $dt$ is the frame difference between $t - 1$ and $t$.

From the transfer matrix $F$, system noise matrix $Q$ , and posterior estimate of covariance at the time $t - 1$, a priori estimate of covariance $P^{\prime}_{t}$ can be here at the time $t$:

6 $$P^{\prime}_{t} = FP_{t - 1} F^{{\text{T}}} + Q$$

The predicted mean vector $z$, the measurement matrix $H$ , and the noise matrix $R$ of the detector allow for the mean error $y$, the Kalman gain $K$ used to estimate the error, the updated covariance $x_{t}$ , and the covariance matrix $P_{t}$:

7 $$y = z - Hx^{\prime}_{t}$$

8 $$K = P^{\prime}_{t} H^{{\text{T}}} (HP^{\prime}_{t} H^{{\text{T}}} + R)^{ - 1}$$

9 $$x_{t} = x^{\prime}_{t} + Ky$$

10 $$P_{t} = (I - KH)P^{\prime}_{t}$$

where $I$ is the unit matrix.

Alternatively, the Mahalanobis distance is considered to be an effective algorithm for measuring the match between the two results, which filters out some of the interference noise. In turn, the Mahalanobis distance $d^{(1)} (n,m)$ between the Kalman filtering prediction result and the target detector result is:

11 $$d^{(1)} (n,m) = (d_{m} - y_{n} )^{{\text{T}}} S_{n}^{ - 1} (d_{m} - y_{n} )$$

Among them, $d_{m}$ is the result of the $m{\text{th}}$ target detector, $y_{n}$ is the result of the $n{\text{th}}$ Kalman filtering prediction, and $S_{n}$ represents the covariance matrix of the $n{\text{th}}$ trajectory. A successful match is indicated when $d^{(1)} (n,m)$ is less than the threshold:

12 $$b_{n,m}^{(1)} = \left\{ {\begin{array}{*{20}c} 0 & {d^{(1)} (n,m) \ge t^{(1)} } \\ 1 & {d^{(1)} (n,m) < t^{(1)} } \\ \end{array} } \right.$$

In (12), $t^{(1)}$ is the quantile at 0.95 of the Chi-square distribution.

**Figure 6:**
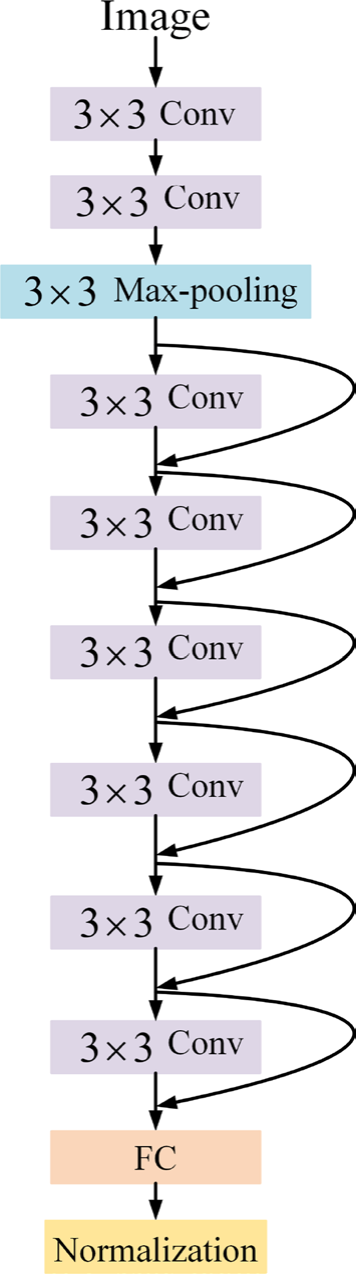
Network structure diagram of the Re-identification model.

However, the motion model of the Kalman-filtered object is uniform, and when there is a variable speed, the results are not reliable if only the Mahalanobis distance is used for the association calculation, so Deepsort also incorporates a re-identification model for extracting appearance features as shown in Table 1 and Fig. 6 is the diagram of this model. The cosine distance enables an effective measure of size similarity between two objects, the minimum cosine distance $d^{(2)} (n,m)$ for the appearance features between the re-identification model and the target detector detection is:

13 $$d^{(2)} (n,m) = \min \{ 1 - \left. {r_{m}^{{\text{T}}} r_{k}^{(n)} } \right|r_{k}^{(n)} \in R_{n} \}$$

where $r_{m}$ is the appearance feature vector of the $m{\text{th}}$ target detector, $r_{k}^{(n)}$ is the $k{\text{th}}$ successful tracking result for the $n{\text{th}}$ trajectory, and $R_{n}$ is the set of 100 latest successful tracking results for the $n{\text{th}}$ trajectory. A successful match is represented when $d^{(2)} (n,m)$ is less than a predetermined threshold $t^{(2)}$:

14 $$b_{n,m}^{(2)} = \left\{ {\begin{array}{*{20}c} 0 & {d^{(2)} (n,m) \ge t^{(2)} } \\ 1 & {d^{(2)} (n,m) < t^{(2)} } \\ \end{array} } \right.$$

**Table 1:** Network structure of the re-identification model.

Layer	Convolution kernel size	Step	Output size
Convolution	$3 \times 3$	1	$32 \times 128 \times 64$
Convolution	$3 \times 3$	1	$32 \times 128 \times 64$
Max-pooling	$3 \times 3$	2	$32 \times 64 \times 32$
Residual	$3 \times 3$	1	$32 \times 64 \times 32$
Residual	$3 \times 3$	1	$32 \times 64 \times 32$
Residual	$3 \times 3$	2	$64 \times 32 \times 16$
Residual	$3 \times 3$	1	$64 \times 32 \times 16$
Residual	$3 \times 3$	2	$128 \times 16 \times 8$
Residual	$3 \times 3$	1	$128 \times 16 \times 8$
Fully connection	–	–	$128$
Normalization	–	–	$128$

In (14), $t^{(2)}$ is the preset threshold.

In Fig. 6, FC is the fully connected layer, Conv is the convolution layer.

The Mahalanobis distance is matched mainly by means of motion information and is more suitable for non-occluded scenes. The cosine distance, on the other hand, is matched by apparent features and is suitable for occluded scenes. So Deepsort calculates a linearly weighted association, whose degree is:

15 $$c_{n,m} = \lambda d^{(1)} (n,m) + (1 - \lambda )d^{(2)} (n,m)$$

where the $\lambda$ is the weight coefficient.

Still, the object that Deepsort is facing is different from that in this paper, so it is challenging to find parameters such as Mahalanobis distance and cosine distance thresholds applicable to spatial target datasets and weight coefficients when both are cascaded. Before judging whether the match is successful, the matching degree between prediction and detection will be calculated by cascade matching sub-module, in which the Mahalanobis distance and cosine distance are linearly added to present the matching degree. The detailed role of this sub-module is shown in Fig. 14.

## Design of spatial dynamic target tracking method

### Dynamic target fusion detection

For the potential ‘ghosts’ and ‘holes’ of the three-frame difference and the difficulty of setting the appropriate threshold by the ViBe-based background subtraction, this section introduces how to design the five-frame difference according to the design idea of the three-frame difference, and how to propose a spatial dynamic target fusion detection method based on the background subtraction based on ViBe.

#### Five-frame difference

**Figure 7:**
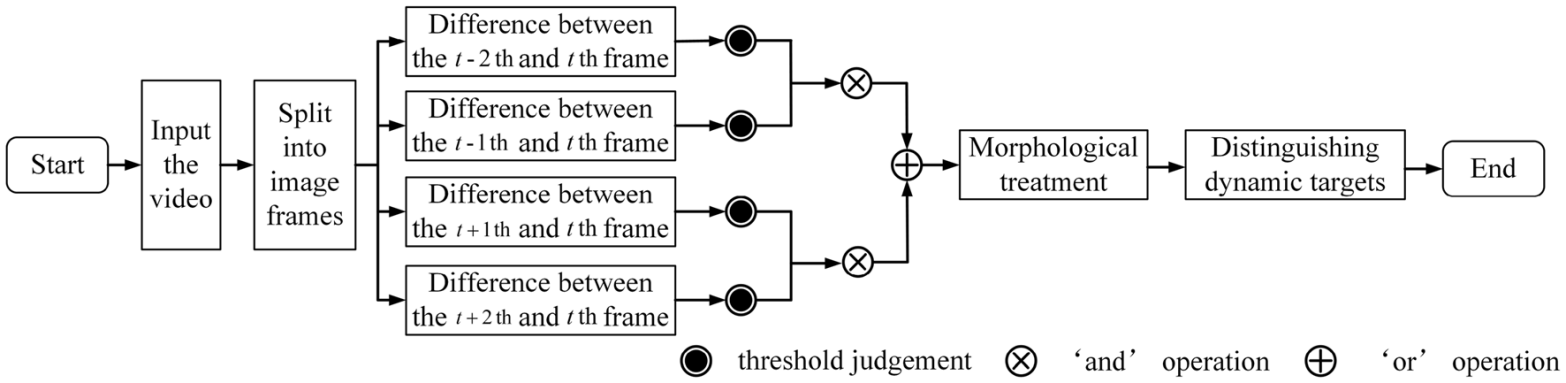
The five-frame difference.

The five-frame difference that can alleviate the ‘holes’ and ‘ghosts’ is designed based on the three-frame difference, and its flow is shown in Fig. 7.

Let the image sequence data of the video split be $I_{1} ,I_{2} ,...,I_{N}$, read the image $I_{t}$ of $t(1 \le t \le N)$ at any moment and the adjacent four-frame images $I_{t - 2}$,$I_{t - 1}$,$I_{t + 1}$,$I_{t + 2}$, the gray value after the grayscale processing of the five-frame image is recorded as $I_{t - 2} (x,y)$,$I_{t - 1} (x,y)$,$I_{t} (x,y)$,$I_{{t{ + }1}} (x,y)$,$I_{{t{ + }2}} (x,y)$, and calculate the absolute value of the gray difference between $I_{t} (x,y)$ and the other four frames of the image in turn, and obtain the difference image $D_{{t{ - }2}} (x,y)$,$D_{{t{ - 1}}} (x,y)$,$D_{t + 1} (x,y)$, and $D_{t + 2} (x,y)$, and the binary images of the difference images are obtained by binarization processing of threshold for difference image, like that $Y_{{t{ - }2}} (x,y)$,$Y_{{t{ - 1}}} (x,y)$,$Y_{t + 1} (x,y)$, and $Y_{t + 2} (x,y)$:

16 $$\begin{gathered} Y_{t - 2} (x,y) = \left\{ {\begin{array}{*{20}c} 1 & {D_{t - 2} (x,y) < T_{1} } \\ 0 & {D_{t - 2} (x,y) \ge T_{1} } \\ \end{array} } \right. \hfill \\ Y_{t - 1} (x,y) = \left\{ {\begin{array}{*{20}c} 1 & {D_{t - 1} (x,y) < T_{2} } \\ 0 & {D_{t - 1} (x,y) \ge T_{2} } \\ \end{array} } \right. \hfill \\ Y_{{t{ + }1}} (x,y) = \left\{ {\begin{array}{*{20}c} 1 & {D_{{t{ + }1}} (x,y) < T_{3} } \\ 0 & {D_{{t{ + }1}} (x,y) \ge T_{3} } \\ \end{array} } \right. \hfill \\ Y_{t + 2} (x,y) = \left\{ {\begin{array}{*{20}c} 1 & {D_{t + 2} (x,y) < T_{4} } \\ 0 & {D_{t + 2} (x,y) \ge T_{4} } \\ \end{array} } \right. \hfill \\ \end{gathered}$$

where $T_{1}$, $T_{2}$, $T_{3}$, $T_{4}$ are dynamic gray difference thresholds adjusted according to the adaptive adjustment of light intensity, and the calculation formula is shown in (17).

17 $$\begin{gathered} T_{1} = \alpha \frac{1}{i \times j}\sum\limits_{x = 0}^{i - 1} {\sum\limits_{y = 0}^{j - 1} {D_{t - 2} (x,y) + T} } \hfill \\ T_{2} = \alpha \frac{1}{i \times j}\sum\limits_{x = 0}^{i - 1} {\sum\limits_{y = 0}^{j - 1} {D_{t - 1} (x,y) + T} } \hfill \\ T_{3} = \alpha \frac{1}{i \times j}\sum\limits_{x = 0}^{i - 1} {\sum\limits_{y = 0}^{j - 1} {D_{t + 1} (x,y) + T} } \hfill \\ T_{4} = \alpha \frac{1}{i \times j}\sum\limits_{x = 0}^{i - 1} {\sum\limits_{y = 0}^{j - 1} {D_{t + 2} (x,y) + T} } \hfill \\ \end{gathered}$$

In (17), $\alpha$ is the adjustment parameter, whose range is the number of pixels in the $2i \times j$-size area, and $T$ is the predetermined binarization threshold.

To suppress ‘ghosts’, the binary images are fused using the logical ‘and’ operation to obtain the fused images $H_{1} (x,y)$ and $H_{2} (x,y)$:

18 $$\begin{gathered} H_{1} (x,y) = Y_{t - 1} (x,y) \cap Y_{t - 2} (x,y) \hfill \\ H_{2} (x,y) = Y_{t + 1} (x,y) \cap Y_{t + 2} (x,y) \hfill \\ \end{gathered}$$

To suppress the ‘holes’, a logical "or" operation is applied to the fused image to obtain the dynamic spatial target image $M_{t} (x,y)$:

19 $$M_{t} (x,y) = H_{1} (x,y) \cup H_{2} (x,y)$$

where $M_{t} (x,y)$ expresses the area of pixels with a grey value of 0 that is the background and 255 that is the motion area of the spatial target.

#### Dynamic target fusion detection

Two problems remain when using the five-frame difference for dynamic target detection: (1) when the dynamic target suddenly stops moving, it may still be present in one or more frames used for difference, resulting in the target still being present in the final detection result; (2) when the dynamic target is moving too slowly, the difference in greyscale between the five frames at the center of the target may not change much and the target may still be internally empty. Internal holes may still be present. The ViBe-based background subtraction can update the background model in time and extract the dynamic target region completely, which can effectively cope with the above problems. At the same time, the dynamic threshold of the five-frame difference can be used to solve the problem that the ViBe-based background subtraction is dependent on the threshold setting. Therefore, this sub-section integrates the designed five-frame difference with the ViBe-based background subtraction to propose a dynamic spatial target detection method.

**Figure 8:**
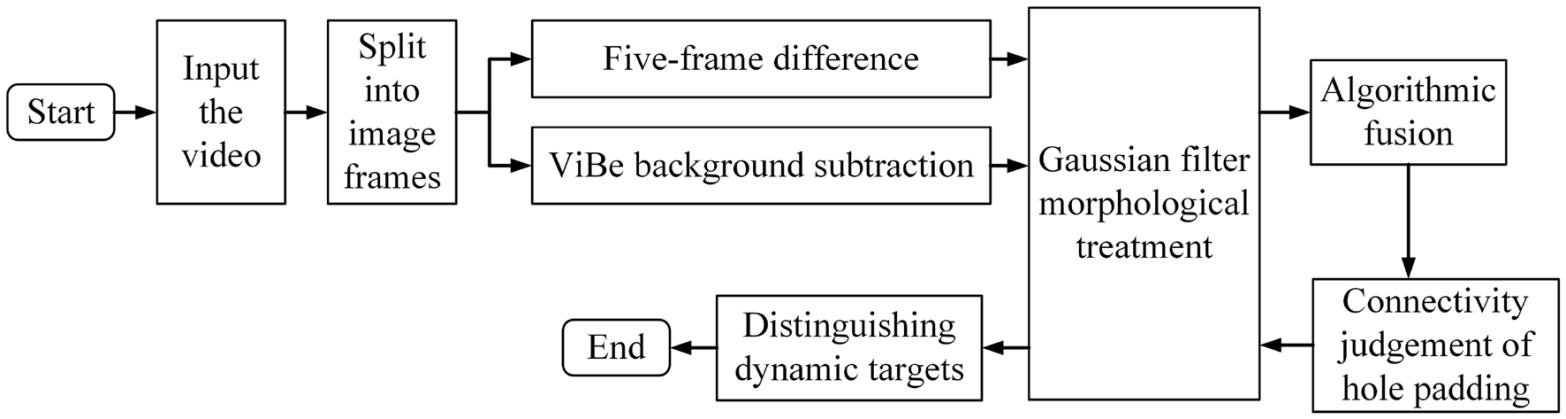
The fusion method of dynamic space target detection.

The method flow is shown in Fig. 8: two algorithms are utilized to detect the spatial target region, the two detection results are processed by Gaussian filtering and morphology, and then fused using the logical ‘and’ operation, and then the fused results are judged for connectivity, and after the ‘hole’ region is filled, its results are processed Gaussian filtering, at which point all the detected targets are the spatial dynamic targets of the motion region.

### Dynamic and online target tracking

As previously mentioned, spatial dim backgrounds have a large impact on vision-based recognition, and to address this issue, this sub-section presents an improved Deepsort detector of YOLOv5s. In addition, there is a self-calibrated spatial target dataset in this paper, and its corresponding appropriate thresholds as well as weight coefficient settings are unfolded in this section.

#### Deepsort detector based on improved YOLOv5s

This sub-section first improves the YOLOv5s to increase its success and accuracy in recognition against dim backgrounds: (1) sharpen and enhance the input image by a target pre-processing method based on DWT; (2) add attention module to the YOLOv5s network model to weaken the background information and enhance the target information. The improved YOLOv5s is then used as a Deepsort detector.

**Figure 9:**
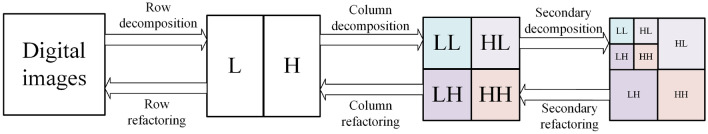
Decomposition and reconstruction of DWT.

Before the DWT, the RGB image will be converted into the HSV image to express the color and achieve a similar visual perception to the human. After conversion, the HSV will be converted back into the RGB for storage. The process of DWT is shown in Fig. 9, the image is decomposed into a series of sub-images with different resolutions through multi-pair high-pass filters and low-pass filters, and the features extracted by the high-pass filter and the compressed image of the low-pass filter are used to enhance the color information, retain the detailed information of the image, and ensure the integrity of the image information.

**Figure 10:**
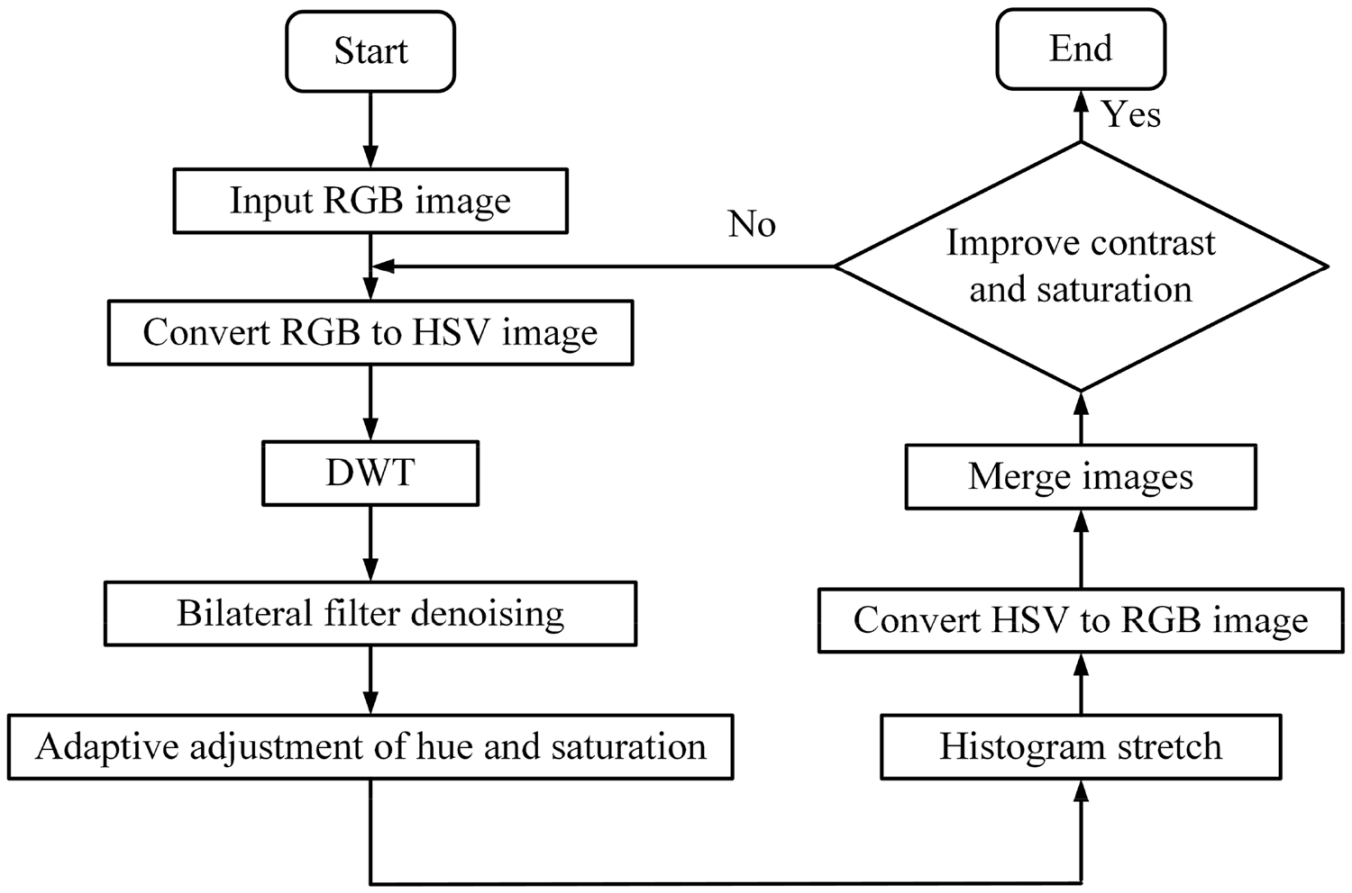
The pretreatment method based on DWT.

The DWT-based detailed preprocessing is presented in Fig. 10. It is worth noting that the DWT is for enhancing the brightness of the HSV image, the bilateral filtering is for removing the noise interference based on effectively retaining a large amount of edge information, the correlation coefficient relationship between the brightness and the three attributes of the HSV image can adjust the hue and saturation adaptively, the histogram stretching is for improving the contrast of the images. Finally, the processed HSV can be converted back to the RGB, in which the high contrast can stay.

**Figure 11:**
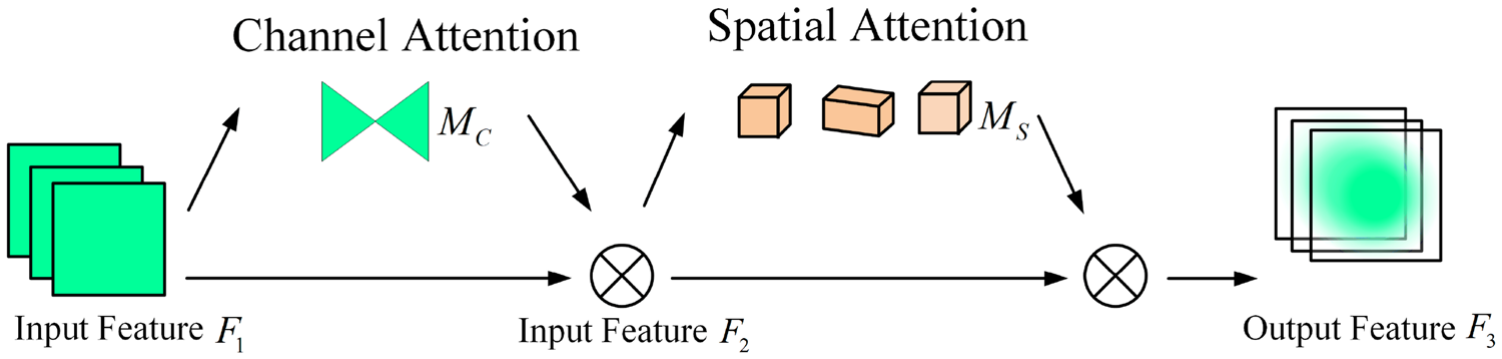
Structure of GAM.

The GAM detailed structure in Fig. 11 is added to the last layer of the Backbone.

**Figure 12:**
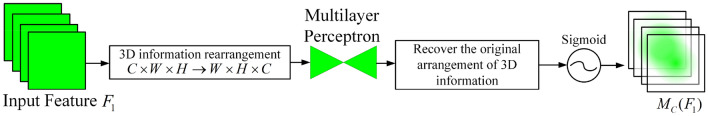
Structure of CAM.

**Figure 13:**
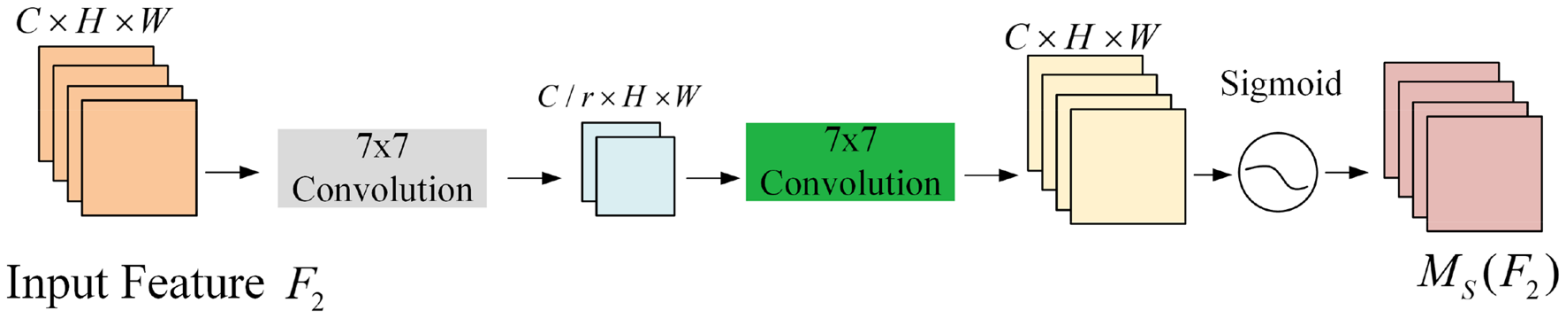
Structure of SAM.

GAM consists of two modules, the Channel Attention Module (CAM) and the Spatial Attention Module (SAM) as shown in Figs. 12 and 13. In the CAM, the three-dimensional information of the image is first rearranged, and then the multilayer perceptron is used to enlarge the cross-dimensional channel space, and all channels are multi-divided by learning the weights of different channels. In the SAM, two convolutional layers are used to fuse the spatial information to make the model pay more attention to the spatial information of the target on the image. Therefore, the use of GAM can improve the information extraction of targets by reducing information reduction and amplifying the global interactive representation, and ignoring the useless black background.

From the results of GAM, CAM, and SAM, it is found that the relationship between the input features $F_{1}$, $F_{2}$ and output features $F_{3}$ is shown as follows:

20 $$F_{2} = M_{c} (F_{1} ) \otimes F_{1}$$

21 $$F_{3} = M_{s} (F_{2} ) \otimes F_{2}$$

where $M_{c}$ is the feature map of the CAM, $M_{s}$ is the feature map of the SAM, and $\otimes$ is tensor multiplication.

#### Dynamic online target tracking based on improved Deepsort

**Figure 14:**
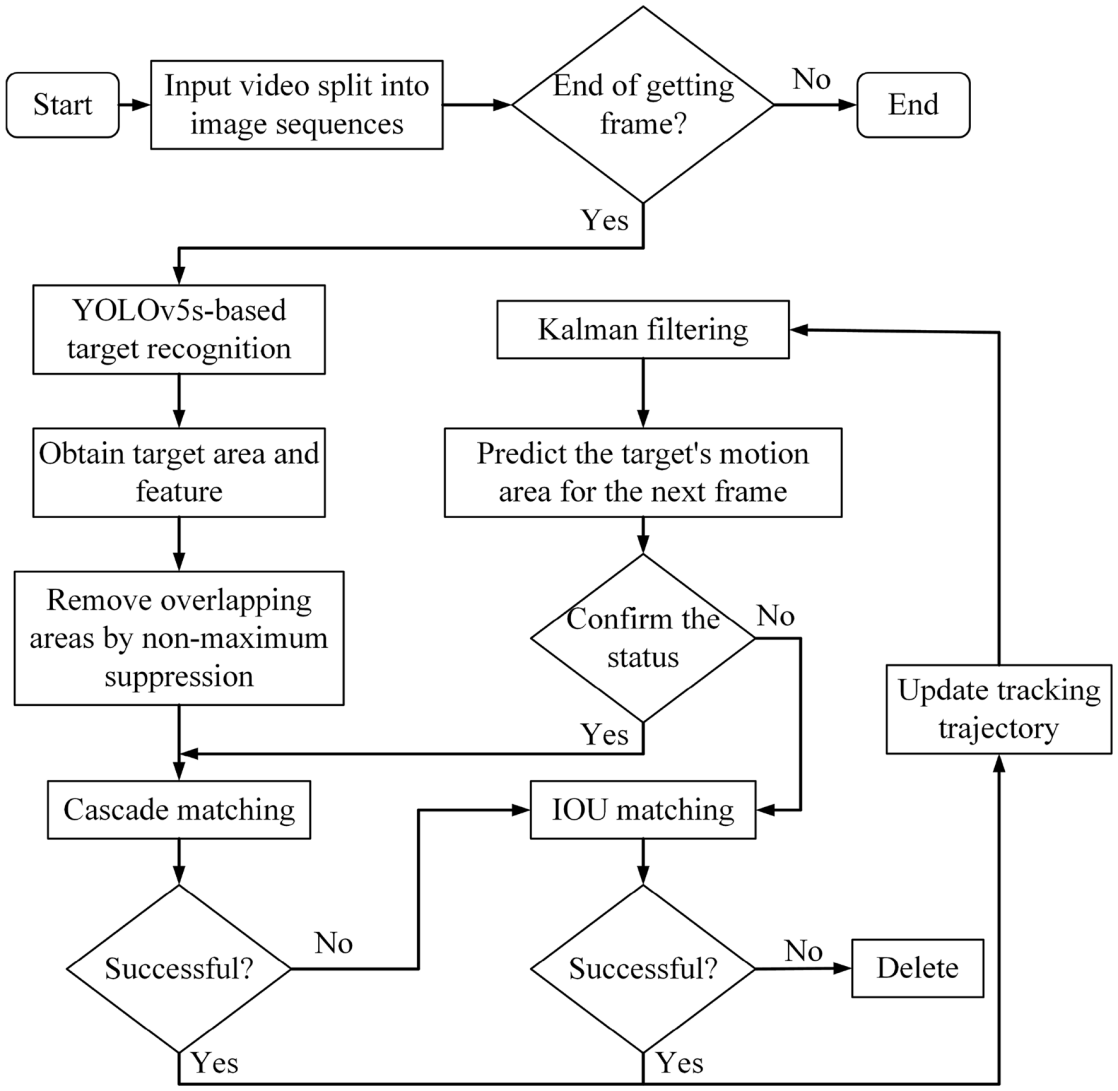
Online tracking of dynamic space targets based on Deepsort.

The flow of dynamic target online tracking based on the improved Deepsort is shown in Fig. 14.

The official tracker parameters provided by Deepsort are described as follows: maximum cosine distance threshold for matching motion information, which ignores the target point if the distance is greater than this threshold; minimum confidence threshold for the target detector for matching appearance features; maximum overlap threshold for non-maximum suppression, which is suppressed if the threshold is set to 1; maximum IOU distance threshold for updating the sequence number, if set too small, the problem of frequent switching of the sequence number is likely to occur; the maximum hit count threshold, the maximum number of frames saved threshold and the maximum lifetime threshold for Kalman filtering update, when the Kalman filtering predicts a new tracking point, if the number of consecutive successful frames of the predicted point is greater than the maximum hit count threshold, the predicted point can be recorded as a new tracking track point, the maximum number of frames saved The threshold is the maximum number of frames to save the latest successful tracking result when calculating the cosine distance. When no successive frames of a target are detected that exceed the maximum lifetime threshold, the target is judged to have disappeared.

The dynamic target online tracking method based on improved Deepsort replaces the target detector with the improved YOLOv5s algorithm designed in this paper. By continuously adjusting the parameters of the tracker for training, it is found that: appropriately decreasing the feature maximum cosine distance threshold and maximum IOU distance threshold can effectively improve the stability of the occlusion during tracking; appropriately increasing the minimum confidence threshold of the target detector can reduce the effect of noise interference; the frequent switching of sequence numbers can be effectively alleviated by appropriately increasing the maximum IOU distance threshold. After continuous experiments, the parameters of the tracker for the online tracking method are finally determined and compared with the parameters of the tracker in the officially downloaded Deepsort as shown in Table 2.

**Table 2:** Deepsort and tracker parameters of the tracking algorithm in this paper.

Parameters	Algorithm of Deepsort	Proposed algorithm
Cosine distance threshold of the Feature maximum	0.2	0.1
Minimum confidence threshold of target detector	0.3	0.5
Maximum overlapping threshold of non-maximum suppression	0.5	1
Maximum IOU distance threshold	0.7	0.5
Maximum number of hits threshold	3	6
Maximum save frame threshold	100	100
Maximum lifetime threshold	70	70

## Simulation and experiment

### Spatial dynamic target detection

**Figure 15:**
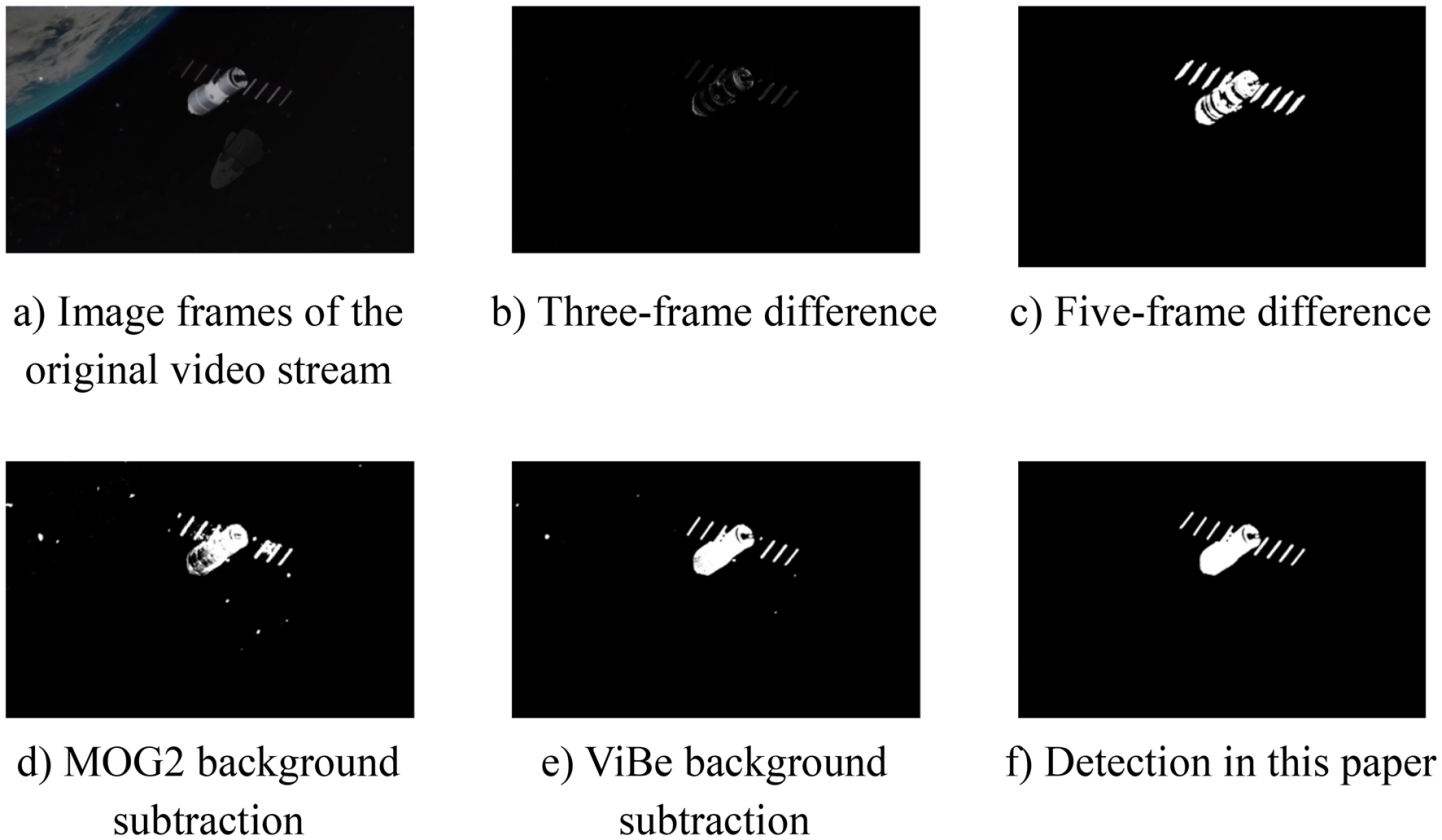
Contrast experiment of dynamic target detection algorithm.

In the laboratory-built algorithm experimental platform, respectively using three-frame difference, five-frame difference, MOG2 background subtraction, ViBe-based background subtraction, dynamic spatial target detection fusion for dynamic target detection of the test video data captured by the vision platform, comparison experiments from the algorithm time consumption and dynamic target motion region identification effect of two dimensions, the comparison results are shown in Table 3 and Fig. 15.

**Table 3:** Time-consuming dynamic target detection.

Algorithm	Three-frame difference	Five-frame difference	MOG2	ViBe	Proposed algorithm
Time-consuming/s	0.012	0.018	0.043	0.031	0.025

**Figure 16:**
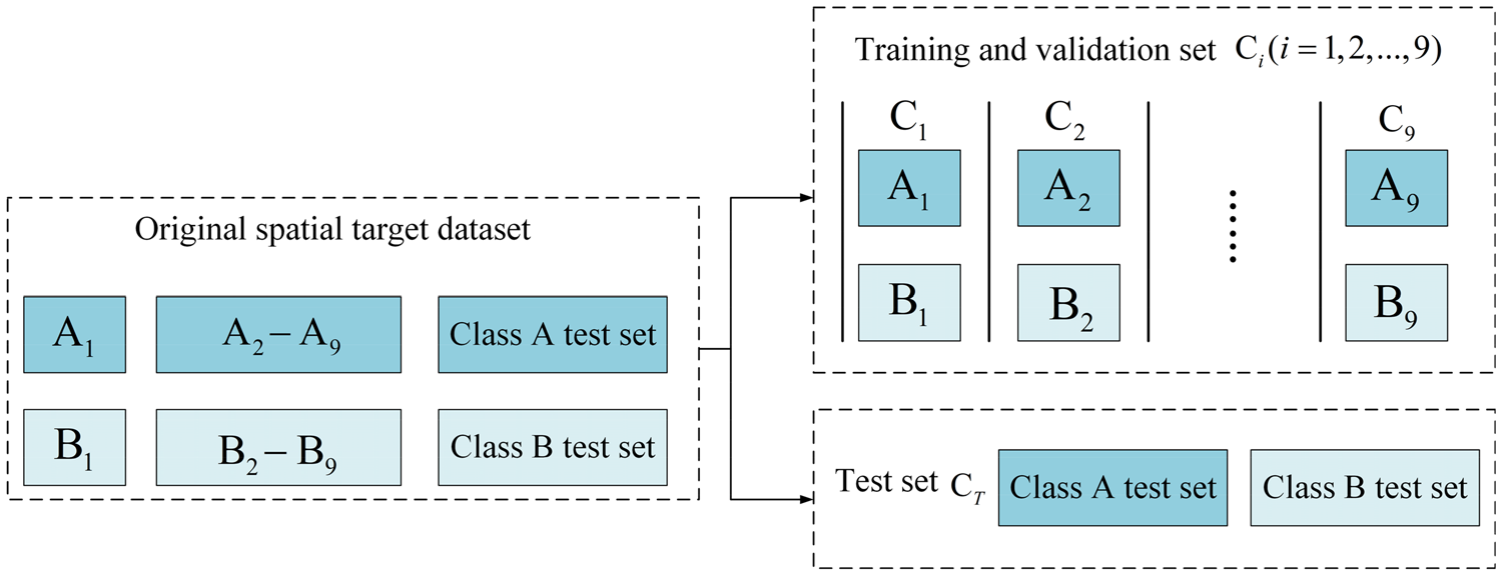
Diagram of ninefold cross-validation experiment dataset division.

**Figure 17:**
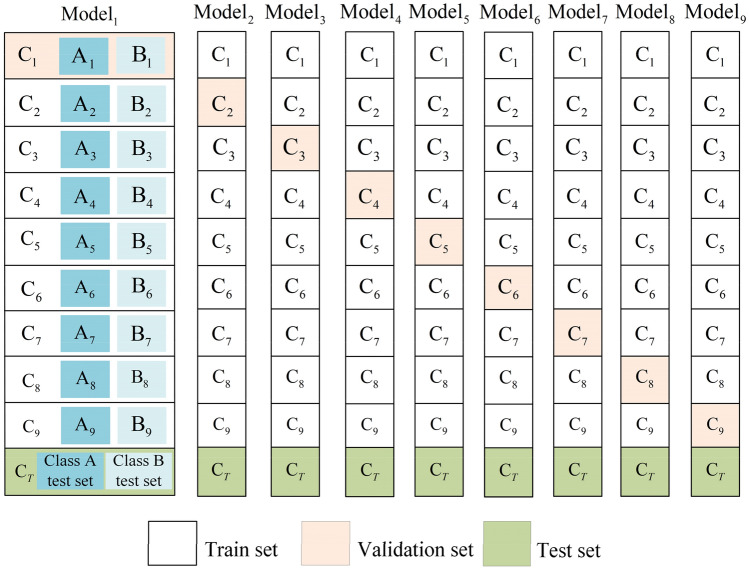
Division of ninefold cross-validation experiment dataset.

With accordance to the results of the comparison experiments, it shows that the detection speed and noise immunity of the three-frame difference and the five-frame difference is good in the dark background of space, and furthermore, the five-frame difference has greatly improved the detection effect compared with the three-frame difference. However, there are still the ‘holes’; The MOG2 background subtraction and the ViBe background subtraction can extract a complete dynamic spatial target motion outline, but the latter is faster and less sensitive to noise than the former. Still, there is the noise caused by the interference of light intensity and other environmental noise; after combining the advantages of the five-frame difference and the ViBe background subtraction, the dynamic spatial target detection fusion proposed in this paper can detect dynamic spatial target motion outline, which is complete and matches the size of the actual spatial target, and it is insensitive to environmental noise factors such as light intensity. Although it is slightly slower in detection speed compared to the five-frame difference, it meets the requirements of real-time for space targets. In summary, this fusion method is with high accuracy and high interference immunity (Figs. 16, 17).

### Experimental results and analysis of tracking dynamic space targets

**Figure 18:**
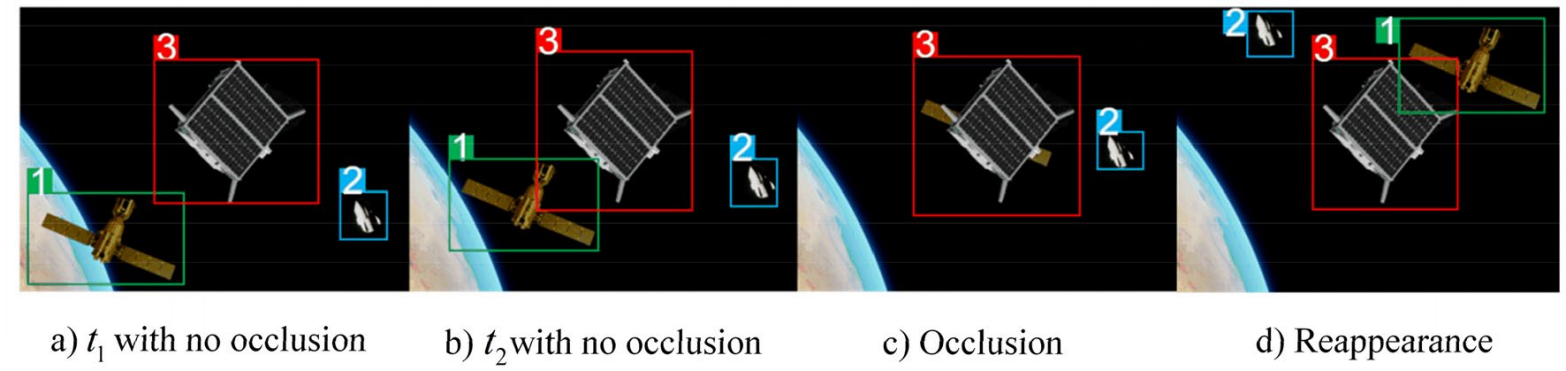
Occlusion experiment of tracking of dynamic space targets.

To verify the performance advantages of the Deepsort-based dynamic space target online tracking method, the official downloadable Deepsort algorithm, the Deepsort algorithm using improved YOLOv5s as target detector, and the nine sets of improved YOLOv5s-based Deepsort algorithms obtained from the cross-validation experiments, in which the best target recognition model is seen as target detectors based on Deepsort target online tracking method for the target tracking experimental task. And the details can be seen in Figs. 16 and 17. In order to verify that the Deepsort-based dynamic space target online tracking method has high stability in the occlusion, the occlusion experiments are conducted in this section, and the experimental results are shown in Fig. 18. (a) and b) show the non-occlusion and normal tracking state, (c) shows the tracking situation when spatial target model 1 is occluded by spatial target model 3 during operation, resulting in the disappearance of model 1, and (d) shows the tracking situation when spatial target model 1 reappears after occlusion from spatial target model 3 for a short time, at which time the sequence number of target model 1 has not changed. The Deepsort-based online tracking method for dynamic spatial targets is thus judged to have good stability for occlusion.

**Figure 19:**
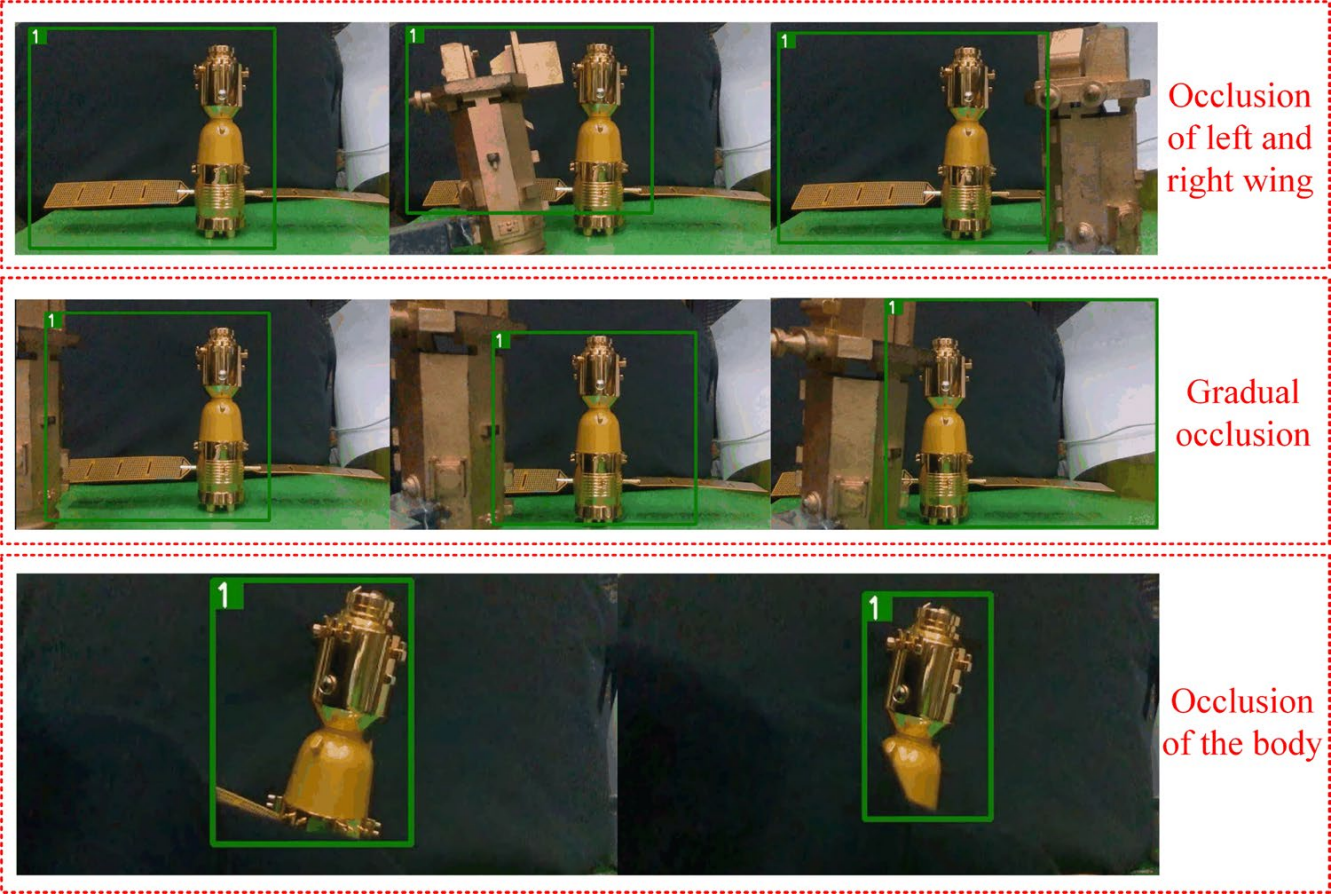
Practical occlusion experiment of targets.

**Figure 20:**
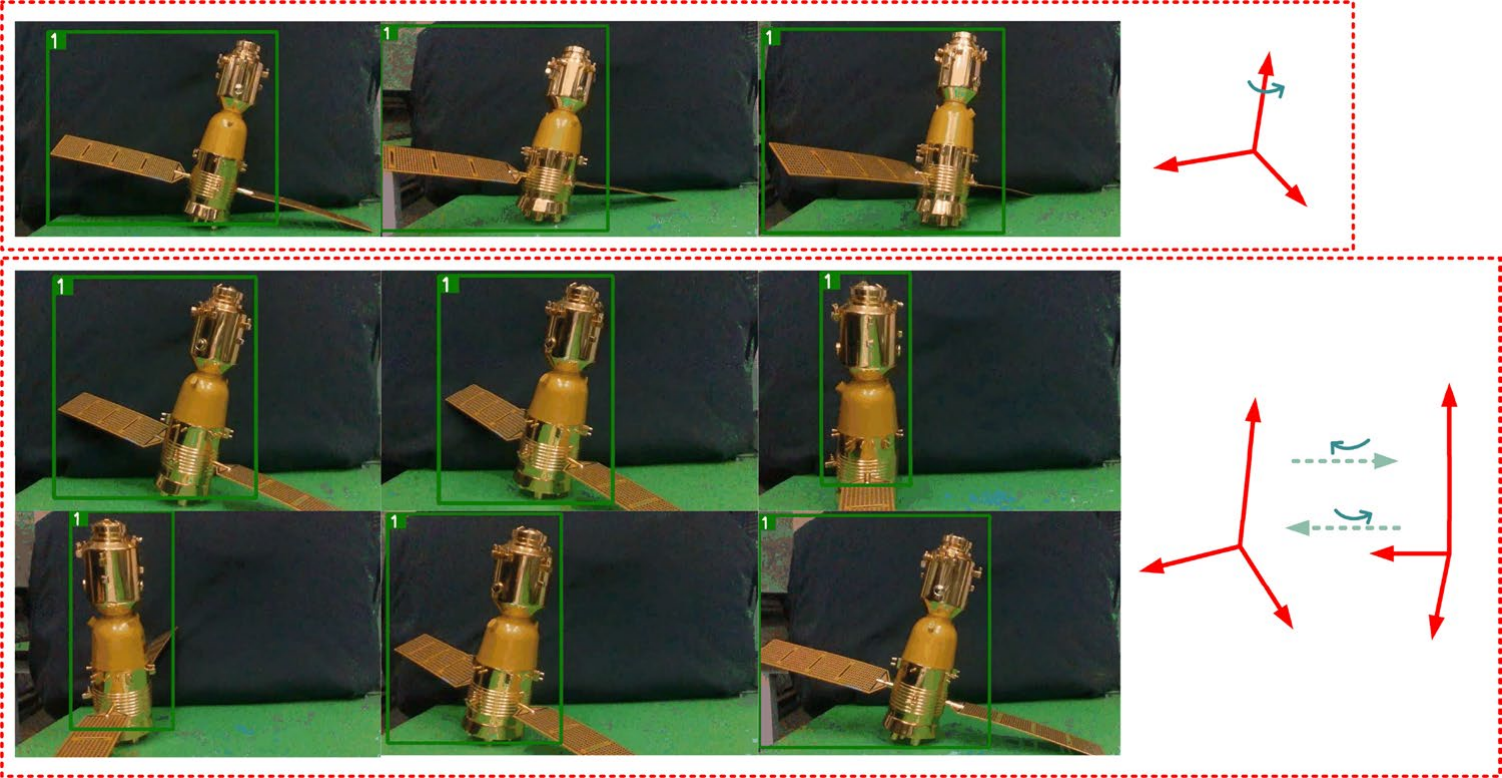
Practical experiment of dynamic targets.

In addition, to verify the effectiveness of this method in practice, simple experiments with actual target occlusion and dynamic operation are conducted. As shown in Figs. 19 and 20, Fig. 19 shows the experiment in the simulated occlusion case and Fig. 20 shows the experiment in the simulated target dynamic state for recognition tracking.

**Figure 21:**
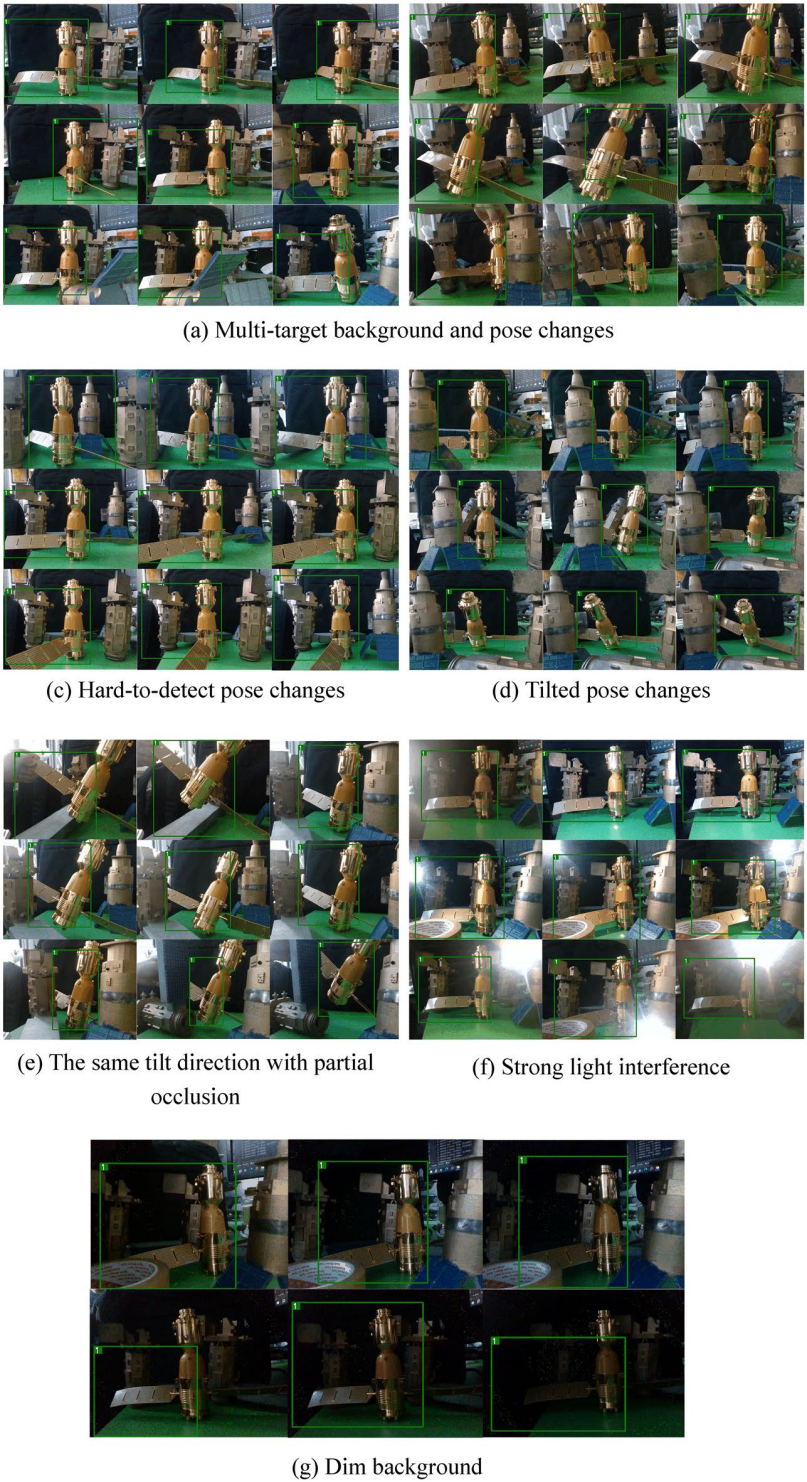
Results of Deepsort &YOLOv5s.

From Figs. 19 to 21, a series of experiments are shown for occluded, and dynamic pose changing targets and for backgrounds with multi-interference targets, strong light and dimness. In particular, the Fig. 21 presents the practical effects of test for the Deepsort&YOLOv5s. And the test results for the other eight models can also be obtained by cross-validation experiment dataset division, which has the explanation in Figs. 16 and 17.

The results are shown in Table 4, where Multiple Object Tracking Accuracy (MOTA) is the percentage of the total number of experiments with no false and missing in the tracking results to the number of all tracked targets, and is used to measure the tracking accuracy; in general, the mismatch between the tracking area of the tracking target and the area where the real target is located is less than the predetermined threshold to judge the success of the tracking, and the multiple object tracking precision is the percentage of the number of tracked targets to the number of all tracking targets, which is used to measure the error of the tracking position.

**Table 4:** Performance comparison of various recognition algorithms based on CNN.

Method	MOTA/%
Original Deepsort	91.13
Deepsort + YOLOv5s	92.93
Deepsort + Model_1_	93.86
Deepsort + Model_2_	93.25
Deepsort + Model_3_	93.93
Deepsort + Model_4_	94.18
Deepsort + Model_5_	94.36
Deepsort + Model_6_	93.58
Deepsort + Model_7_	93.69
Deepsort + Model_8_	94.41
Deepsort + Model_9_	93.97

Based on the experimental results, it can be seen that the mean MOTA value of the tracking using the nine sets of best identification models obtained in the cross-validation experiments of the dataset as target detectors is about 93.88%. Clearly, the proposed Deepsort-based dynamic space target online tracking can improve tracking accuracy.

## Conclusion

For space dynamic target tracking scenarios with dim, dynamic environments and multiple targets in occlusion, it is a great challenge for visual recognition and tracking techniques. This paper proposes an online tracking method based on a five-frame difference with a depth association metric online real-time tracking algorithm, which incorporates DWT pre-processing for images, a GAM aiming at enhancing target information, five-frame difference, and a fusion of ViBe-based background subtraction and Deepsort online tracking, and ultimately achieves first identification and then tracking of dynamic targets.

Specifically, this paper designs a five-frame difference along the three-frame difference and fuses itself with background subtraction to achieve improved accuracy and interference immunity. Based on the presence of potential other problems in the image, the image quality and model are enhanced by means of DWT and GAM to the Deepsort detector. Additionally, Deepsort is improved for the occlusion from multiple targets and loss of target information, and reasonable hyperparameters and thresholds are set for the customized dataset to improve the real-time and accuracy of the tracking. The effectiveness and superiority of the improved method are verified by datasets cross-validation experiments and other learning methods. The simulation experimental results show that the proposed dynamic space target tracking method can achieve stable tracking of all targets under occlusion and improve the tracking precision to 93.88%. Finally, experiments are conducted with the physical depth camera D435i on background interference and occlusion situations, and they show the effectiveness and superiority of the proposed identification and tracking algorithm in the face of the strong light and occlusion. Even though the potential dramatic change in pose and complex occlusion is not considered. Future work will explore the further improvement of occlusion and pose changes for the application in space capture and attitude estimation.

## Data Availability

The datasets generated during and/or analysed during the current study are available from the corresponding author on reasonable request.
